# Using mixtures of biological samples as process controls for RNA-sequencing experiments

**DOI:** 10.1186/s12864-015-1912-7

**Published:** 2015-09-17

**Authors:** Jerod Parsons, Sarah Munro, P. Scott Pine, Jennifer McDaniel, Michele Mehaffey, Marc Salit

**Affiliations:** Material Measurement Laboratory, National Institute of Standards and Technology, 100 Bureau Drive, Gaithersburg, MD 20899 USA; Leidos Biomedical Research Inc., P.O. Box B Bldg 428, Frederick, MD 21702 USA; Department of Bioengineering, Stanford University, 443 Via Ortega, Stanford, CA 94305 USA

**Keywords:** RNA sequencing, RNA-seq, Gene expression, Mixture deconvolution, Expression deconvolution, Process control, Spike-in control, ERCC

## Abstract

**Background:**

Genome-scale “-omics” measurements are challenging to benchmark due to the enormous variety of unique biological molecules involved. Mixtures of previously-characterized samples can be used to benchmark repeatability and reproducibility using component proportions as truth for the measurement. We describe and evaluate experiments characterizing the performance of RNA-sequencing (RNA-Seq) measurements, and discuss cases where mixtures can serve as effective process controls.

**Results:**

We apply a linear model to total RNA mixture samples in RNA-seq experiments. This model provides a context for performance benchmarking. The parameters of the model fit to experimental results can be evaluated to assess bias and variability of the measurement of a mixture. A linear model describes the behavior of mixture expression measures and provides a context for performance benchmarking. Residuals from fitting the model to experimental data can be used as a metric for evaluating the effect that an individual step in an experimental process has on the linear response function and precision of the underlying measurement while identifying signals affected by interference from other sources. Effective benchmarking requires well-defined mixtures, which for RNA-Seq requires knowledge of the post-enrichment ‘target RNA’ content of the individual total RNA components. We demonstrate and evaluate an experimental method suitable for use in genome-scale process control and lay out a method utilizing spike-in controls to determine enriched RNA content of total RNA in samples.

**Conclusions:**

Genome-scale process controls can be derived from mixtures. These controls relate prior knowledge of individual components to a complex mixture, allowing assessment of measurement performance. The target RNA fraction accounts for differential selection of RNA out of variable total RNA samples. Spike-in controls can be utilized to measure this relationship between target RNA content and input total RNA. Our mixture analysis method also enables estimation of the proportions of an unknown mixture, even when component-specific markers are not previously known, whenever pure components are measured alongside the mixture.

**Electronic supplementary material:**

The online version of this article (doi:10.1186/s12864-015-1912-7) contains supplementary material, which is available to authorized users.

## Background

Measurement assurance for genome-scale measurements is challenged by the impracticality of creating a sample containing known quantities of tens of thousands of components, such as the RNA transcripts measured in an RNA-seq experiment. Deep sequencing of cellular RNA can generate vast quantities of gene expression information, yet measurement biases have been identified at nearly every step of the library preparation process [1–4].

As RNA-sequencing expression data expands from discovery into clinical applications, the sources and magnitudes of bias and variability must be carefully understood and quantified. The basic units of expression in sequencing, such as transcripts per million reads (TPM) or fragments per kilobase per million reads (FPKM), are still undergoing revision [5, 6]. Even when using comparable units, it is rarely possible to directly compare gene expression values reported by different labs, on different instruments, or frequently just on different days [6–8], unless special care is taken to use uniform samples and protocols. Identifying the presence and variation of biases in a measurement process over time requires a standard to be used for process control. The regular use of a process control can help determine the most-appropriate protocol and analysis methods, demonstrating that the measuement process accurately represents the true changes in the underlying sample.

Ideally, a measurement process is linear and possesses a known precision. A linear measurement process shows an increase in signal proportional to an increase in the object being measured. It is also helpful if measured signal is additive, arising only from a single source. Precision consists of repeatability and reproducibility, defined as the degree of closeness in multiple measurements made by a single user and the closeness between multiple labs, respectively. We show that mixtures can demonstrate that a measurement’s response function is linear and of high specificity (free of interference or cross talk) while measuring its variability and precision. Properly constructed mixture samples can be used to correct for systematic measurement errors, provide ongoing monitoring of performance, serve as a tool for interlaboratory comparison, and create a context for evaluating batch effects, protocols, and informatic analyses.

There are two known approaches to creating useful genome-scale standards. One is the creation of a limited number of external spike-in controls, such as those designed by the External RNA Controls Consortium (ERCC), which were created for microarrays and have been applied to next-gen sequencing [9–11]. A second approach utilizes mixtures of previously characterized samples in defined ratios, and has also been applied to microarrays [12–14] but has not been utilized in other genome-scale measurements. Using these types of standards provides confidence in the ability of a test to detect both positive and negative results, determining the limits of that detection.

Mixtures can serve as a test that applies to each of the tens of thousands of transcripts in a sample’s transcriptome. Linearity of the measurement response can be demonstrated based on the fundamental understanding that a mixture is a linear combination of its components. Previous work with mixtures in microarrays [12–14] utilized an arbitrary 10-fold “selectivity” cutoff to evaluate the linear dynamic range of microarray measurements and understand the variability of these measurements. The arbitrary selectivity cutoff in previous work prevents the identification of interference, as any genes affected by interference would be filtered by the stringent selectivity cutoff.

Using known mixture compositions, predicted values can be calculated based on the assumption that the measurement response is linear. Deviation of the observed values from the model-predicted value is an indication of bias in the measurement. Systematic biases could be introduced by sample preparation, signal processing, interference from related or mis-annotated genes, or sampling variation. Signal arising from off-target molecules, such as a closely related transcript, can cause false positive results and result in a lowered specificity. Mixture samples can provide information about the measurement sensitivity, specificity, repeatability, reproducibility, dynamic range, and limit of detection.

Determining the relative contributions to gene expression of individual components within mixtures of biological states has received some attention in clinical research, where biopsies and other patient samples are often mixtures. The process of resolving gene expression signals introduced by each individual component of a mixture [13–23] has been used to account for tumor heterogeneity and to separate whole blood samples into individual cell types. These procedures often separate mixture components based on a subset of genes forming a signature that varies uniquely between components. These deconvolution methods have been used [24–27] to develop high-resolution tumor expression signatures from imperfect biological samples [28, 29] and differentiate between cell-type-frequency changes and per-cell gene expression changes [17, 30]. Many of these methods can determine mixture component types by using a linear model where mixture expression is treated as a combination of expression signatures.

None of these methods corrects for RNA enrichment. Different cell types express different total amounts of RNA of varying types, confounding estimates of cell type proportion made based on the quantification of total RNA [31]. Others have introduced the concept of a biological scaling factor [32, 33] to compensate for variation in the RNA content of cells, including the use of spike-in controls to determine this factor. The enrichment of subclasses of RNA from total RNA (as in polyA selection) adds a bias to the experiment due to the different abundance of RNA classes between cell types.

We assess linear response, specificity, and accuracy of genome-scale measurements using mixtures. In the process, we demonstrate that linear models can be used to separate these mixtures into the proper components. We were mindful that while our mixtures were of total RNA, the sequencing process enriches for RNA subclasses, and that differential enrichment is an important factor when interpreting results. We anticipate that a mixture-based approach to measurement assurance is highly generalizable to many types of mixtures and can be extended to the wide variety of genome-scale measurements, including but not limited to proteomic and metabolomic measurements.

## Results

**Fig. 1:**
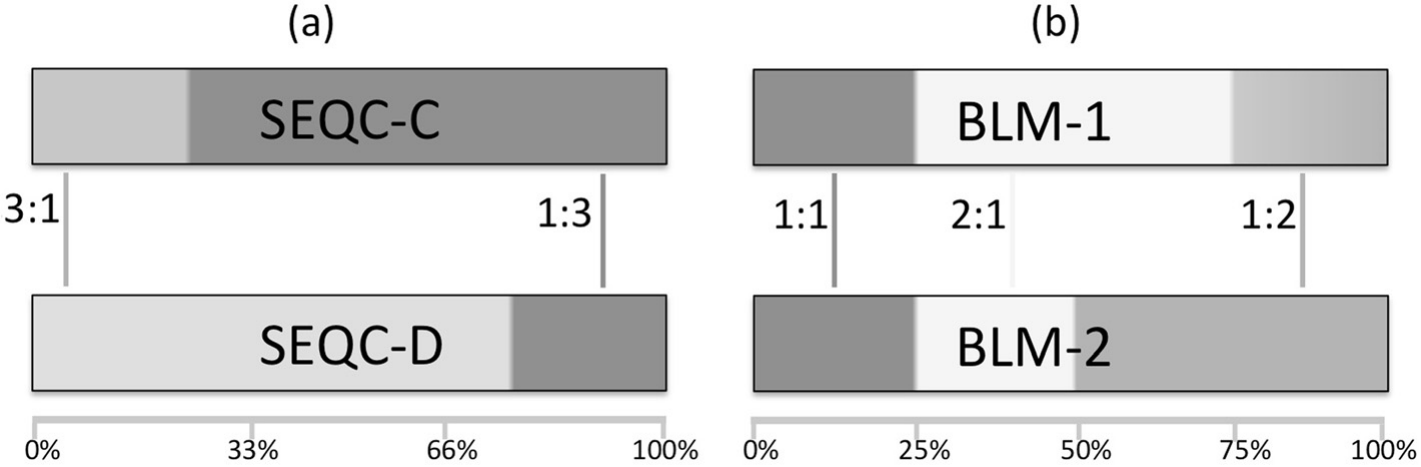
RNA samples used in this study. RNA isolated from pure tissues is used to generate pairs of mixtures used in two separate experiments. **a** Two SEQC mixtures (SEQC-C and SEQC-D) are built from two components (SEQC-A and SEQC-B). **b** Two BLM mixtures (BLM-1 and BLM-2) are built from three components. The SEQC-B component (HBRR) is from the same source as the Brain BLM component. Per-sample target ratios of tissue proportion between mixtures are shown

To assess measurement parameters of genome-scale transcriptome data, we analyzed two RNA-seq experiments measuring synthetic mixtures of commercially available human total RNA samples (Fig. 1) [13, 14, 34]. First, we analyzed data generated as part of the Sequencing Quality Control Consortium (SEQC) project [34, 35], which contained two mixture samples as part of their inter laboratory experiment. In this study, the 9 laboratories sequenced the following samples: Universal Human Reference RNA spiked with ERCC ExFold RNA Spike-in Mix 1 (SEQC-A), Human Brain Reference RNA spiked with ERCC ExFold RNA Spike-in Mix 2 (SEQC-B) and two mixtures of SEQC-A and SEQC-B (SEQC-C and SEQC-D) with mixture compositions C = 3A + 1B and D = 1A + 3B.

In a second experiment, which we call BLM, we generated multiple libraries of two mixtures (BLM-1 and BLM-2) composed of total RNA isolated from human brain (B, the same RNA as SEQC-B), liver (L), and muscle (M) tissue were measured for this study. These two mixtures were made with component proportions of 1B:1L:2M and 1B:2L:1M. The total RNA of each individual tissue were also sequenced as single component samples to provide an expression signature for each tissue. ERCC spike-in control RNAs [12] prepared by NIST were added to the BLM mixtures and individual components. Two spike-in control pools were designed with ratiometric differences in the concentration of individual ERCC spike-ins. The multiple libraries of BLM-1 and BLM-2 mixtures were spiked with either of these pools at one of three (high, low, medium) concentrations. As expected based on the mixture designs, ERCCs spiked-in equally yielded similar expression signal, while signal from ERCCs spiked differentially into multiple subpools was at ratios corresponding to the designed fold changes. Poisson sampling at the lower expression levels results in increased dispersion about the expected ratio [36].

These mixtures were designed to have a defined expression signal ratio between them. For example, if the measurement response were linear and unbiased, the signal in the SEQC-C sample would be exactly 1/4 the signal of SEQC-B plus 3/4 the signal from SEQC-A due to the design of the mixture. However, these total RNA mixtures went through RNA-seq library preparation by polyA selection, which purposely removes certain classes of RNA, such as ribosomal RNA, from the sample. As the resulting sequence data comes from only the selected subset, the fraction of which can be different from sample to sample, a correction for this differential enrichment must be applied to accurately reflect the experimental process and allow the model to return the designed ratios of expression between mixtures (Additional file 1: Figure S1).

### Linear model-based analysis of genome-scale gene expression

We observed that mixture expression is a linear combination of the component samples and the mixture proportions of each component. Equation 1 describes the relationship between signal in the mixtures and signal in the constituent samples. A mixture *M* (two per dataset in this study) is composed of a number of named components *C* (“B”,”L”, and”M” in the Brain/Liver/Muscle mixture or “A” and “B” in the SEQC dataset), with each component comprising a proportion of the mixture. Φ_C_. _i,M_ is the expression signal arising from a particular gene/transcript *i* in mixture *M*.

1 $$ {}_{\mathrm{i},\mathrm{M}}{=}_{\mathrm{i},\mathrm{C}}\times {\varPhi}_{\mathrm{C},\mathrm{M}} $$

This study uses four mixtures of the same general form:

$$ \begin{array}{l}{}_{\mathrm{i},\mathrm{B}\mathrm{L}\mathrm{M}1}{=}_{\mathrm{i},\mathrm{B}}\times {\varPhi}_{\mathrm{B},1}{+}_{\mathrm{i},\mathrm{L}}\times {\varPhi}_{\mathrm{L},1}{+}_{\mathrm{i},\mathrm{M}}\times {\varPhi}_{\mathrm{M},1}\hfill \\ {}{}_{\mathrm{i},\mathrm{B}\mathrm{L}\mathrm{M}2}{=}_{\mathrm{i},\mathrm{B}}\times {\varPhi}_{\mathrm{B},2}{+}_{\mathrm{i},\mathrm{L}}\times {\varPhi}_{\mathrm{L},2}{+}_{\mathrm{i},\mathrm{M}}\times {\varPhi}_{\mathrm{M},2}\hfill \\ {}{}_{\mathrm{i},\mathrm{SEQC}-\mathrm{C}}{=}_{\mathrm{i},\mathrm{SEQC}-\mathrm{A}}\times {\varPhi}_{\mathrm{A},\mathrm{C}}{+}_{\mathrm{i},\mathrm{SEQC}-\mathrm{B}}\times {\varPhi}_{\mathrm{B},\mathrm{C}}\hfill \\ {}{}_{\mathrm{i},\mathrm{SEQC}-\mathrm{D}}{=}_{\mathrm{i},\mathrm{SEQC}-\mathrm{A}}\times {\varPhi}_{\mathrm{A},\mathrm{D}}{+}_{\mathrm{i},\mathrm{SEQC}-\mathrm{B}}\times {\varPhi}_{\mathrm{B},\mathrm{D}}\hfill \end{array} $$

These mixtures were made of total RNA, while the expression signal (sequencing reads) arises only from the enriched RNA. As the fraction of the total RNA mass that matches the enrichment criteria varies between cell types, the enrichment of total RNA introduces a bias. Additional file 1: Figure S1 shows the offset from the expected ratios of tissue-specific and ERCC RNA caused by this bias. We correct the specific equations for the enrichment fraction by multiplying each component by a factor ρ. This factor corresponds to the fraction of measured RNA compared to the mass of total RNA in each mixture. ρ_*C*_ is defined as the fraction of measured RNA per unit total RNA in component *C*.

Including this factor, the BLM1 mixture equation becomes Eq. 2:

2 $$ {}_{\mathrm{i},\mathrm{B}\mathrm{L}\mathrm{M}1}{=}_{\mathrm{i},\mathrm{B}}\times {\varPhi}_{\mathrm{B},1}\times {\rho}_{\mathrm{B}}{+}_{\mathrm{i},\mathrm{L}}\times {\varPhi}_{\mathrm{L},1}\times {\rho}_{\mathrm{L}}{+}_{\mathrm{i},\mathrm{M}}\times {\varPhi}_{\mathrm{M},1}\times {\rho}_{\mathrm{M}} $$

There are a few approaches that have been described to measure ρ. One study directly measured the post-selection RNA content between SEQC-A and SEQC-B samples [37]. Another described the use of trimmed mean of log expression ratios (TMM) [32] to measure a biological scaling factor based on enriched RNA directly from RNA-seq data. These TMM-derived factors have been shown to be an appropriate measure in cases where there is no global expression level change (such as the SEQC mixtures), but are not applicable if there are global expression changes (such as in the BLM mixtures) [33].

The ρ factor can be determined using spiked-in RNA [33] as sample reads per microgram of total RNA divided by spike-in reads per microgram of spike-in RNA. This factor utilizes the differential enrichment between polyadenylated spike-in RNA and total RNA, which is only partly composed of polyadenylated RNA.

**Fig. 2:**
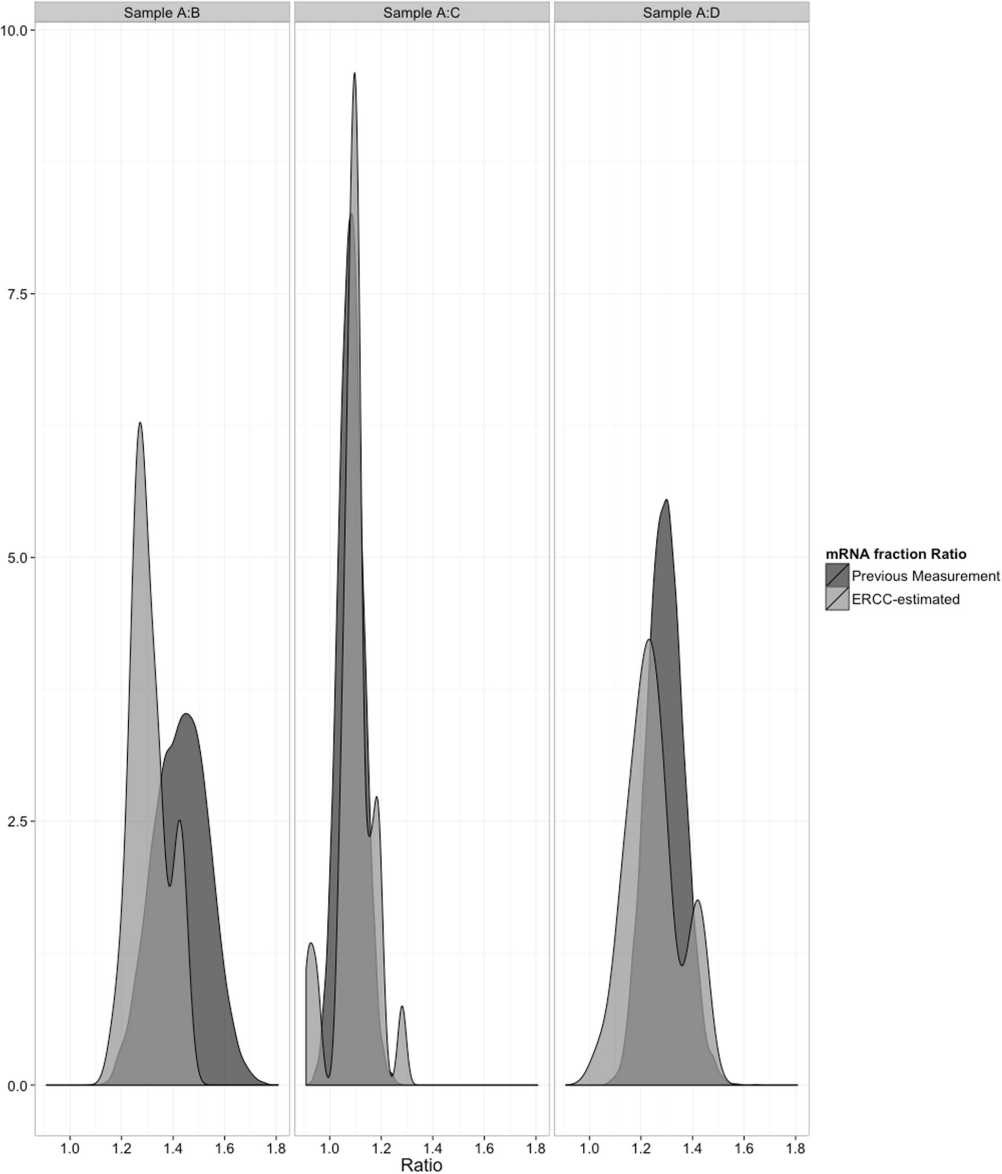
Distributions of empirical (light) and ERCC-estimated (dark) enrichment ratios between SEQC samples A:B, A:C, and A:D. The empirical distribution was simulated from a normal distribution with means of 2.87 and 2.003 and standard deviations of 0.095 and 0.124 for samples A and B, as reported previously [37]. The ERCC-estimated values were calculated from Equation 3. Individual labs’ RNA enrichment varied inside a narrow range, yielding discrete peaks in the distribution for some outlying labs

Figure 2 compares the distributions of spike-in estimated rho factor ratios across the SEQC samples compared to the direct measurement of poly-A enriched RNA made previously [37]. While the ρ factors do not directly measure the polyA content of a sample due to relatively inefficient but consistent polyA capture of the spike-in RNA, ratiometric measurements of pairs of samples have distributions that are similar to that of a normal distribution with parameters based on the previous enrichment measurements of SEQC-A and SEQC-B. Additionally, the expected equalities of ρ_C_ = ρ_A_*.75+ ρ_B_*.25 and ρ_D_ = ρ_A_*.25+ ρ_B_*.75 hold true to within 5 % of ρ_A_, indicating that the enriched RNA content of a mixture is a linear combination of the enriched RNA content of its components. Additionally, solving the system of BLM equations only for the enrichment fractions (inputting the known proportion values) yields very similar enrichment fractions to those calculated from spiked-in RNA, leading us to be confident in these measurements.

The target RNA fraction ρ is a property of an individual RNA sample and can be affected by any sample manipulation - chief among them the polyA selection step in sample preparation. For replicates within a single polyA-selected SEQC experimental run, the ρ of a mix varies slightly, likely due to fluctuations in efficiency of polyA selection. (Additional file 2: Table S1) It is also important to note that FPKM units should not be used to calculate the enrichment fraction (Additional file 3: Figure S2), as the FPKM derivation [6] includes a term coupling sample abundance to spike abundance.

### Mixture analysis models recapitulate known mixture proportions

**Fig. 3:**
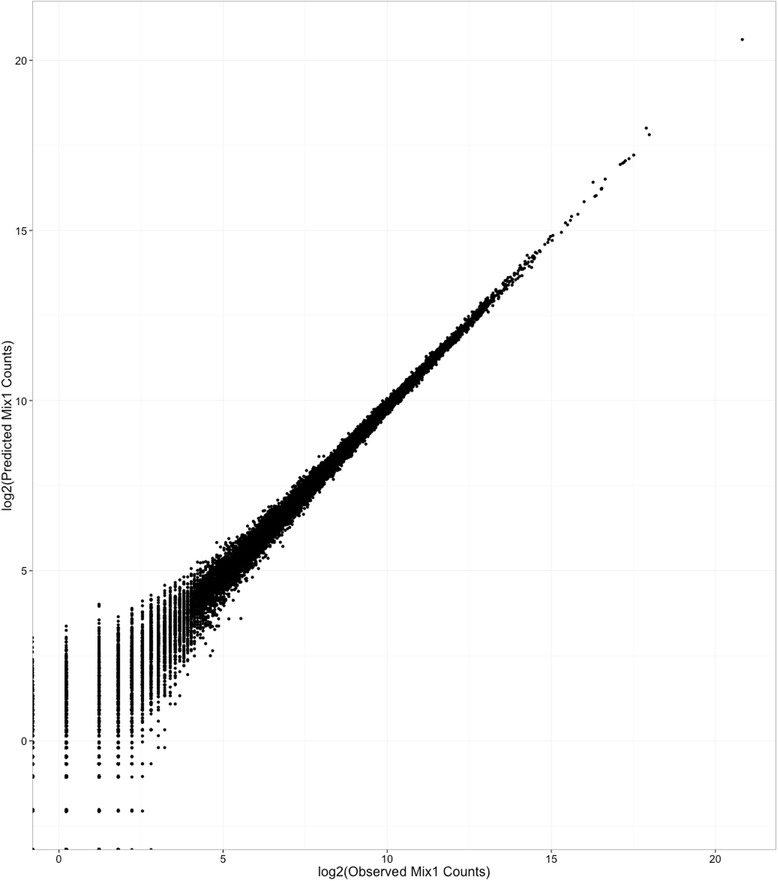
Comparison of observed and predicted counts. Observed BLM mix 1 counts (x) are plotted against predicted BLM mix 1 counts (y). Predicted counts are calculated using eq. 2. Counts are on the log2 scale

**Fig. 4:**
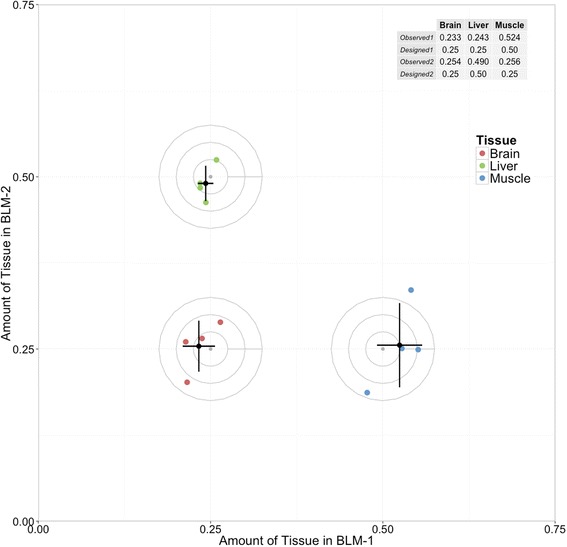
Accuracy of model-derived BLM mix estimates. The grey center point is the nominal ‘truth’ ratio in which the samples were mixed. Concentric circles with radius at multiples of 0.025 are added to visually clarify distance from the center point. Colored points depict mixture proportion *(*Φ*)* estimates generated from measurements of 4 replicate libraries. Black points are the mean of the replicates. Error bars show one standard deviation of the four replicate measures

**Fig. 5:**
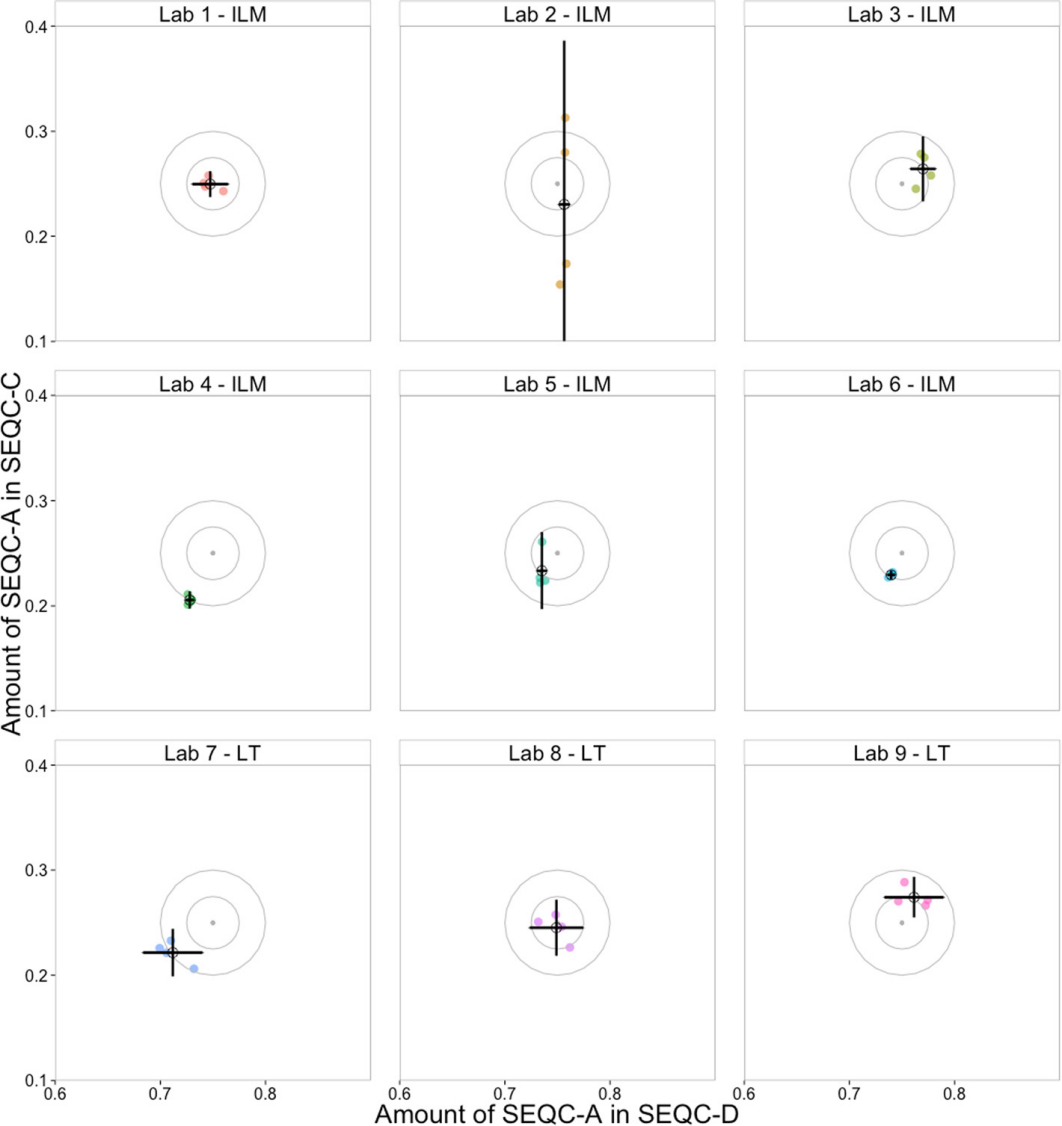
Mixture proportion (Φ) estimates for samples A in SEQC-C and SEQC-D. The mean (black hollow circle) and standard deviation (error bars) of four individual replicates (colored) of the Φ estimate for each sample are shown. The nominal mixture proportions are grey points at the center of the target. Circles centered at that nominal ratio with radii in multiples of .025 are included to more easily identify magnitude of total error. LT and ILM tags indicate the manufacturer of the sequencer used at each lab (Life Technologies and Illumina, respectively). Deviations from the target indicate process variability, instrument bias, or errors brought about in these labs

To demonstrate the accuracy of this analytical framework of mixtures, the mixture proportions Φ_*BLM*_ were recalculated for the BLM mixtures BLM-1 and BLM-2. The ρ values and the sequencing expression data X_i_ were used to solve for the mixture proportions Φ_*BLM*_ by linear regression to the mixture equation. Figure 3 shows that the experimentally observed counts are highly correlated (R^2 = 0.996) to the equation-solved counts X_i_ for each transcript. Figure 4 shows the Φ_*BLM*_ values at which residuals were minimized for the two mixtures for each replicate sample in each laboratory. Estimates of the three component proportions in the two mixtures are consistent with the designed 25:25:50 and 25:50:25 proportions in the two BLM mixtures. Figure 5 shows that the designed proportions of SEQC mixtures across each of nine labs can also be calculated by this equation, returning the 75:25 and 25:75 proportions for mixes C and D, with some variability between labs. Eq. 1, which lacks correction for enrichment fraction, does not return the designed ratios (Additional file 4: Figure S3).

### Linear model-predicted mixture counts are equivalent to replicate measures

In studies by the SEQC [34], differential expression between replicate samples was utilized to evaluate measurement performance based on the hypothesis that the control samples used in the study had no true differences between replicates. We created pseudo-replicate predicted count values from the single component samples for use in benchmarking. These simulated mixtures were built based on the measured mixture expression and the true mixture proportions.

**Fig. 6:**
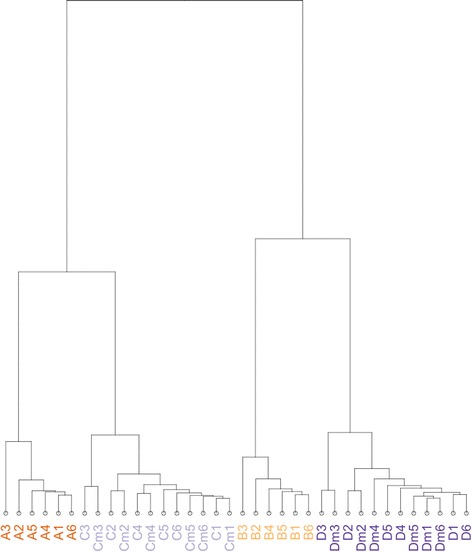
Clustering of Expression measures in 4 SEQC samples and 2 *in-silico* replicate samples across participating sites: The close agreement between modeled (Cm, Dm) counts and actual counts (A, B, C, D) at sites numbered 1–6 supports the validity of assumptions used to model Cm and Dm counts. Euclidian distance measures between samples show that the various samples are of greater distance from one another, while the *in-silico* modeled samples are most similar to the correct corresponding sample

Figure 6 shows a dendrogram of the distance between actual mixture expression and predicted expression counts of SEQC samples. The four base samples A, B, C, and D are most distant from one another, reflecting the biological differences between the samples. Samples A and C are more closely related, as C consists of 75 % A and 25 % B. Modeled pseudo-replicate samples Cm and Dm across each of the six SEQC sites are no more different than cross-lab replicates of the C and D data, indicating that building the model for mixture C from components A and B does not introduce significant variability. This supports the treatment of modeled mixtures as replicate measurements expected to have no true differential expression from the mixture samples. Any detected differential expression between a mixture and its predicted expression values is indicative of a bias in the measurement process. In the BLM or SEQC datasets, differential expression was detected only in the ribosomal RNA genes (NR_003286.2, NR_003287.2, NR_023363.1). This detected differential expression reflects the sample to sample variance in rRNA depletion.

## Discussion

Mixtures of biological samples can be useful as process controls for measurements with linear response functions. A mixture can be treated as linear combination of its components. Two experimental datasets with known mixture parameters were used to test the linearity of RNA-seq measurements. In RNA-seq, the enrichment of the total RNA mixture components by polyA selection must be accounted for, as the sequencing experiment measures only the RNA which passes this enrichment filter.

Mixtures with either known or unknown proportions can be analyzed. If mixture proportion information is known a priori, genome-scale data can be used as a process control to test the repeatability and sensitivity of measurements by comparing observed and expected measures. Alternatively, if the mixture proportions are an unknown and desired parameter, expression measures from the mixture in combination with the single components can be used to experimentally determine the mixture proportions. This application can be valuable in un-mixing biological mixtures, including clinical mixtures, cell cultures, and xenografts [24–29]. While target RNA fraction correction is required for RNA-sequencing measurements, the general mixture model is theoretically applicable to any measurement with a linear response function.

Mixtures can provide measurement process assurance to a sequencing experiment. Using mixture samples alongside pure samples, one can demonstrate the reproducibility and sensitivity of genome-scale RNA, protein, as well as metabolite measurements. The main goal of this type of mixture analysis is to create a known ratio value by which the measurement characteristics of an experiment can be assessed. While an experiment’s measurement of this known ratio is not sufficient to prove the validity of the measurement, it is a necessary condition, and any deviations are indicative of bias.

We demonstrate process control usage of mixtures by comparing the nine SEQC sites. Figure 5 shows a summary plot of the estimated component fractions for each sample. The dispersion and bias of the points from the target value give an indication of the overall process accuracy. Within this set of labs there are easily discernible changes, which could indicate process errors. Site 1 looks strong – there is no bias, and a modest and regular level of dispersion. In site 2 the dispersion of component C is exaggerated, suggestive of an issue in the handling of that particular sample. Site 4 has less dispersion than site 1, but has introduced a bias. Site 7 is from a completely different sequencing instrument, and shows that there is similar dispersion to the previous instrument, with a bit of a bias. However, site 8 shows that this bias does not occur in every run. This comparison of SEQC sites shows that even these summary plots can detect differences between runs. It is for this reason that we suggest the use of mixtures as process controls for RNA-seq experiments. Comparing the dispersion and bias of your measurement against the known truth of mixtures as you make changes to your experimental process allows you to evaluate the effect of these changes on the measurement quality. Table 1 describes several types of changes that can be evaluated in this way.

**Table 1:** Example use cases for process control mixtures

User	Sample design	Time to use	Benefit
Technology and Experimental Protocol Developers	Mix 2–3 components of biological interest appropriate for use to evaluate the experimental protocol/technology	When validating experimental protocols and/or technology platforms	Demonstrate the ability of a protocol and/or technology platform to consistently measure transcripts of interest
Core Labs	Repeated measures of highly-available samples	After changes in technician, prototol, reagents, or technology platform, etc.	Show internal consistency of output and linearity of measurements
			Identify biases
			Understand dispersion
Informaticians	Relevant samples, public data (eg: SEQC, this study)	When comparing informatics analysis tools or developing new tools	Use non-simulated benchmark datasets to determine how accurate results are.

While we demonstrate mixture analysis with two specific samples, the analysis is generalizable to any number or type of mixture components. Any mixture split into known individual components can be measured in this way. For example, a clinical researcher may have three samples of interest from healthy, chronically diseased and acutely diseased sources. A mixture of these three cell types would provide confidence in the measurements made on the three samples individually by verifying the repeatability of that measurement. It can also provide a benchmark sample to assess comparability over space and time. These mixtures can detect biases introduced by batch effects, operator effects, sample mislabeling, and technical artifacts while evaluating the variability of the measurement. Mixture samples with known proportions can help determine experimental reproducibility and discover technical artifacts introduced by the measurement process by comparison of the expected to observed proportions.

With this analytical model, end users and core facilities can use known mixtures as a process control to track changes in measurement quality whenever changes to the experimental process are made. By including a predefined mixture, cross-sample comparisons can be made to demonstrate the internal consistency of measurements made using any new experimental technique, kit, or downstream analysis tool. In this way, there is some assurance that changes in experimental protocol have not affected measurement reproducibility. Residuals from modeled counts can be used as a metric to evaluate the magnitude of effect an experimental process has on the linearity and precision of underlying measurements.

In addition to gaining an understanding of the measurement process using the benchmarking workflow, unknown samples can be collected and studied to determine the relative proportion of known components. Proportions of components can be determined even in the absence of any type-specific markers, given measurable differences in expression between the cell types.

Resolving the composition of mixtures has proven useful in determining the purity of cell lines or proportions of heterogeneous cells, in identifying interesting cellular contaminants such as partially differentiated cells, and understanding clinical samples containing mixed cell types. In contrast to approaches using transgene expression [38], the mixture model described here can evaluate tissue sample purity without focusing on a handful of tissue-specific genes, marker genes, or transgenes. We expect mixed-sample RNA to be useful in regulatory applications, where a demonstration that a therapeutic stem-cell mixture has a specific composition may be key to ensuring safety and efficacy [39].

### Spike-in controls measure post-enrichment RNA content of samples

In addition to providing limit of detection and cross-experiment comparison characterizations of a dataset, spike-in controls can be used in mixture samples to determine the enriched RNA fraction of cells. Enrichment fraction is a critical parameter for comparing samples that do not have identical total RNA content. This is most relevant to cells with variable global expression [31], including comparisons across and within cell cycle, tissues, and developmental states [40]. Enrichment fraction is also critical in single cell gene expression studies, where lysis efficiency and total RNA content can vary greatly from cell to cell.

We demonstrate that the ERCC controls can be used as an estimator of enriched RNA content within samples. Of note, the SEQC study [34] results showed a large degree of variation in sample sequencing library preparation even at the same site, but that the sequencing library replicates prepared at a single site and then sequenced at multiple laboratories resulted in very consistent measurements between sites. Variation in library prep is primarily due to variability in RNA enrichment, and is the primary source of variability in spike-in controls [41, 36].

There are many methods used to determine component gene expression profiles from mixture samples. To the best of our knowledge, our method is the only one that accounts for RNA enrichment as calculated via spike-in controls. When comparing samples of variable RNA content, bias arises when that variability is not accounted for. We describe a straightforward method for measuring the enrichment of target RNA in RNA-seq samples using spike-in RNA. We show that enrichment-corrected deconvolution of two mixture datasets returns the best approximation of known mixture proportions (Figs. 4 and 5), demonstrating suitability for solving unknown mixtures of known components.

Previous methods used to determine the composition of RNA-seq mixtures make inaccurate estimates of mixture proportion in the BLM sample where the enrichment fractions vary substantially between mixture components. These methods are nearer to true values in the SEQC sample, where the RNA content difference between samples is less significant, but all estimates are improved by incorporating enrichment fraction measurement (Additional file 4: Figure S3).

### Recommendations for use

Control mixtures most easily demonstrate that an experimental process is linear and internally consistent, and can track the changes in variability over time. A first experiment with a new process should utilize these controls to demonstrate the reproducibility of measurements between single component and mixture samples. Subsequently, changes to the process can be evaluated by comparing the model residuals before and after the change. For example, a lab interested in changing from a total RNA measurement to a messenger RNA measurement may wish to evaluate if this change had any effect on sequencing output. The change in the sum of residuals between these two different experiments would allow a global comparison, while the change in residuals of individual genes may highlight a set of genes, which become inconsistently biased between experiments. Table 1 shows three potential use cases for mixtures used as process control.

### Limitations

Although mean mixture proportion values returned from a linear combination of mixture components approximate the nominal mixture proportion in both measured samples, the increased variability of the muscle estimate in the BLM mixture (error bars, Fig. 4) suggests that there is a lower limit to being able to determine low-abundance mixture components. Due to a lower target RNA fraction in muscle, that component of the BLM mix was as low as 10 % of sequenced RNA in BLM-2. It may be possible to determine lower-proportion mixture components with confidence, but this study did not generate the required data to do so.

Our estimation of targeted RNA fraction is imperfect; an assumption of the model we built is that the enrichment proportion is constant between replicates of the same sample. Additional file 2: Table S1 shows that the actual enrichment varies by as much as 5 % from library to library. This variability is a source of error in our model. The variability in enrichment is likely due to batch effects in the polyA selection process. This hypothesis is reinforced by the prevalence of non-polyA transcripts incorrectly called as differentially expressed between mixture replicates. Another limitation is that the targeted RNA fraction is based on total RNA mass, rather than per cell. Researchers interested in the relative proportions of cells in a mixture will additionally need a measurement of average mass per cell. The sequencing technology and library preparation methods used in these experiments also added limitations to the experiments. These are described in Additional file 5: Note S1.

## Conclusions

We demonstrate the linear response function and specificity of RNA-sequencing measurements using mixtures of biological samples. We recommend the use of such mixtures as benchmarks to characterize the repeatability and reproducibility of experiments. Spike-in controls can be used to calculate the measured RNA content of total RNA mixtures, compensating for biases introduced by polyA enrichment or similar RNA enrichment techniques. Our method creates a framework for using mixtures in measurement process control and corrects for biases introduced by RNA selection. Correction for differential enrichment improves the accuracy of mixture proportion determination in RNA-seq experiments.

Benchmarking genome-scale measurements using mixed samples will remain useful even after the era of short-read sequencing is over. Answering the biological question of “what types of cells are in the mixture I’m sequencing?” requires more information than even a perfect transcriptome reconstruction could provide. The biological and measurement value added by mixed samples are demonstrated here to be platform-independent. We anticipate that mixtures can provide the same measurement assurance to protein and metabolite measurements. Confidence in the reproducibility of measurement and understanding the components in complex biological samples will always be a staple of quality science.

## Methods

### Library preparation

For the BLM experiment, Human Brain Reference RNA, Human Liver Total RNA, and Human Skeletal Muscle Total RNA were purchased from Ambion. Human RNA tissues were purchased from Ambion. Ambion certifies that all human derived materials have been prepared from tissue obtained with consent from a fully informed donor or a member of the donor’s family.

This purified RNA was quantified by absorbance on a NanoDrop 1000, mixed in the specified proportions, then spiked with ERCC RNA transcribed from NIST SRM 2374. For Illumina sequencing, the Illumina TruSeq protocol was followed. HiSeq runs generated 100 + 100 bp paired-end reads. Solid 5500 sequencing followed the Life Technologies Whole Transcriptome protocol, yielding 75 + 35 bp paired-end reads. Spike-in composition and amounts are included in the data submission to ENA.

### Quantitation and data normalization

BLM gene counts were based on raw count data quantified using HTSeqCounts [40] based on a variety of genome and transcriptome references [42–45] after mapping reads to the genome with Tophat [46]. Raw counts were then normalized using the upper quartile method implemented in EdgeR [36]. Additional file 4: Figure S3 utilizes RSEM [47]. HTSeq-counts version 0.5.4 was run with options to deal with non-stranded reads in the intersection-nonempty mode. The SEQC data used are available as count tables from GEO GSE47774. Counts used in the final data analysis are from the UCSC “all genes” reference modified to add ERCC controls.

#### Calculating unknown mixture estimates

The relative abundance of components in unknown mixtures were calculated by first observing the mean target RNA fraction for the neat components across replicates. The count data in the mixture was set as the response, predicted by the count data from the individual components modified by the enrichment fraction, as based on the mixture equations. An example R script ‘generalmixturesolver’ is provided at http://github.com/usnistgov/mixtureprocesscontrol as a supplemental file to clarify this procedure.

### Availability of data and materials

The SEQC data is available from GEO GSE47774. [http://www.ncbi.nlm.nih.gov/geo/query/acc.cgi?acc=GSE47774].

The BLM data is available from the European Nucleotide Archive, PRJEB8231. [http://www.ebi.ac.uk/ena/data/view/PRJEB8231].

Figure code, count tables, and example scripts are available on https://github.com/usnistgov/mixtureprocesscontrol.

### Ethics

No ethics approval was required from an ethics committee for the study.

## Additional files

Additional file 1: Figure S1. Bland-Altman log-ratio(M) - log average (A) plots comparing gene expression in BLM-1 to BLM-2, which were mixed with a designed ratio of 1:1 brain RNA, 2:1 muscle RNA and 1:2 liver RNA. Points representing gene expression values for genes expressed at 5-fold greater levels in a specific tissue are colored based on the tissue in which they are selectively expressed. Non-tissue selective RNA are omitted for clarity. Library size normalization scales all libraries to a common total number of counts, while upper quartile normalization scales to the 75^th^ percentile of the counts for each library. None of these normalizations accurately reflects the designed ratio of transcripts between samples. (PNG 473 kb) Additional file 2: Figure S2. The effect of using FPKM units. Estimates of enrichment fraction (light points are calculated using count values, dark points using FPKM values) result in a relatively poor solution to the mixture proportion. Both data types are taken from the same RSEM output. (DOC 29 kb) Additional file 3: Figure S3. Mixture proportions returned by a simple model (Eq. 1, blue squares), by an enrichment-corrected model (ρ*-corrected* mixture equations, green triangles) and by the DeconRNASeq package [36] (red circles) on SEQC data. Lab # - LT and - ILM indicate the manufacturer of the sequencer used at each participating lab (Life Technologies and Illumina, respectively).DeconRNASeq implements the same general idea, but lacks enrichment fraction correction. In the SEQC data, there is a relatively small enriched fraction difference between samples, but significant improvements are nevertheless achieved by correcting for the enriched fraction. The mean distance from true value across all SEQC labs is 0.052 (Simple model), 0.033(enrichment-corrected), and 0.048 (DeconRNASeq). Error bars represent the SD of four independent libraries from the same RNA source. (PNG 78 kb) Additional file 4: Table S1. Enrichment fraction (ρ) calculations as a function of spike amount. Spike mass is accounted for in the enrichment calculation. The spike-ins varied by amount (“u” or “d” samples) and content (pools ‘a’ or ‘b’) in both tissue mixtures (1 and. 2). Calculated enrichment fractions vary by +/- 6 % across these 10 BLM mixtures, showing that the calculation is robust to spike-in mass and content. Enrichment calculations for the ERCC pools must account for the 3-plex nature of the mixes. The shown ratios are for the subset of spike-ins which are present at a 1:1 ratio in each sample. (PNG 119 kb) Additional file 5: Note S1. RNA-seq is capable of making transcript isoform-specific measurements. However, long reads of high depth are required to adequately differentiate between isoforms. Investigations of isoform-level measurements from the BLM dataset, which utilized 75 × 35bp paired-end reads on the 5500 and 100 × 100 bp paired-end reads on the HiSeq, showed that while the model is extensible towards such measurements, the reduced mean read counts make transcript isoform-level expression measurements less precise due to shorter read length and lower sequencing depth. 92 % of genes were modeled to within 1 log2 unit of the measured value, while only 85 % of transcripts were [38]. (DOC 28 kb)

## Abbreviations

ERCC: External RNA control consortium
TPM: Transcripts per million
FPKM: Fragments per Kilobase per million mapped reads
mRNA: messenger RNA
