# Supplementary figures and images for: Using mixtures of biological samples as process controls for RNA-sequencing experiments
Source: BMC Genomics. 2015 Sep 17;16(1):708. doi: 10.1186/s12864-015-1912-7 (PMC4574543; doi:10.1186/s12864-015-1912-7)

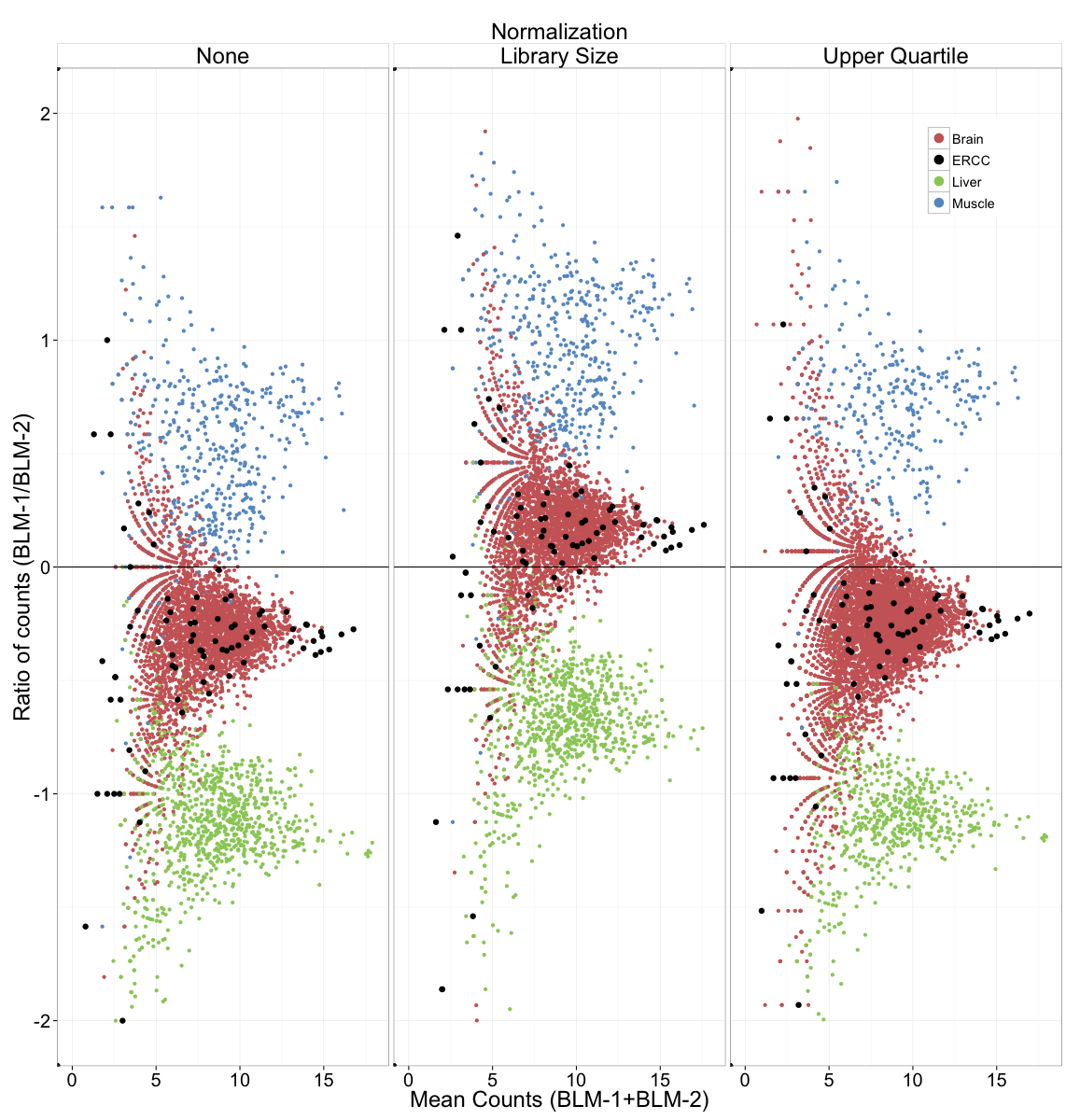

Supplement: Additional file 1: Figure S1. — Bland-Altman log-ratio(M) - log average (A) plots comparing gene expression in BLM-1 to BLM-2, which were mixed with a designed ratio of 1:1 brain RNA, 2:1 muscle RNA and 1:2 liver RNA. Points representing gene expression values for genes expressed at 5-fold greater levels in a specific tissue are colored based on the tissue in which they are selectively expressed. Non-tissue selective RNA are omitted for clarity. Library size normalization scales all libraries to a common total number of counts, while upper quartile normalization scales to the 75th percentile of the counts for each library. None of these normalizations accurately reflects the designed ratio of transcripts between samples. (PNG 473 kb) [file 12864_2015_1912_MOESM1_ESM.png]

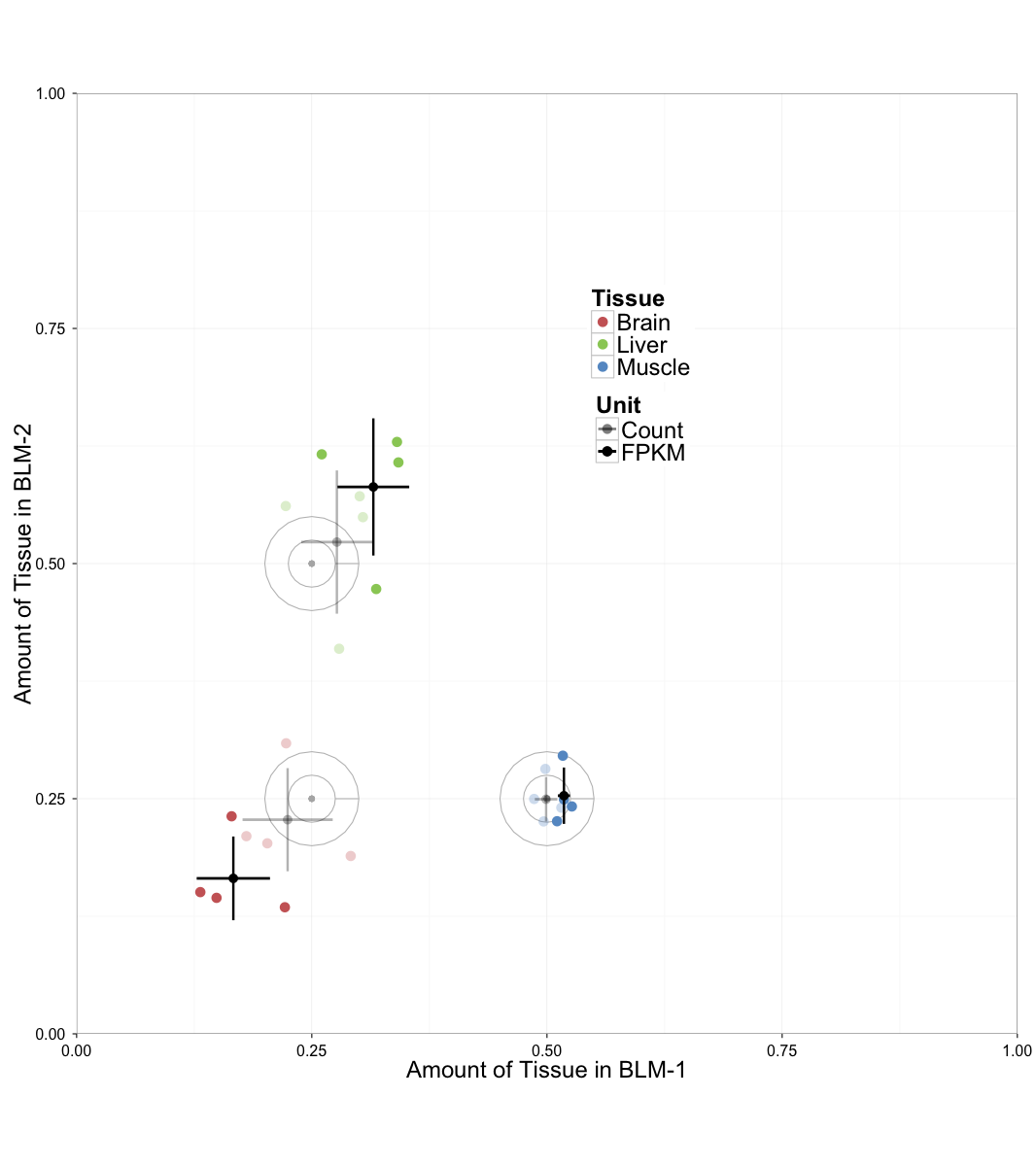

Supplement: Additional file 3: Figure S3. — Mixture proportions returned by a simple model (Eq. 1, blue squares), by an enrichment-corrected model (ρ-corrected mixture equations, green triangles) and by the DeconRNASeq package [36] (red circles) on SEQC data. Lab # - LT and - ILM indicate the manufacturer of the sequencer used at each participating lab (Life Technologies and Illumina, respectively).DeconRNASeq implements the same general idea, but lacks enrichment fraction correction. In the SEQC data, there is a relatively small enriched fraction difference between samples, but significant improvements are nevertheless achieved by correcting for the enriched fraction. The mean distance from true value across all SEQC labs is 0.052 (Simple model), 0.033(enrichment-corrected), and 0.048 (DeconRNASeq). Error bars represent the SD of four independent libraries from the same RNA source. (PNG 78 kb) [file 12864_2015_1912_MOESM3_ESM.png]

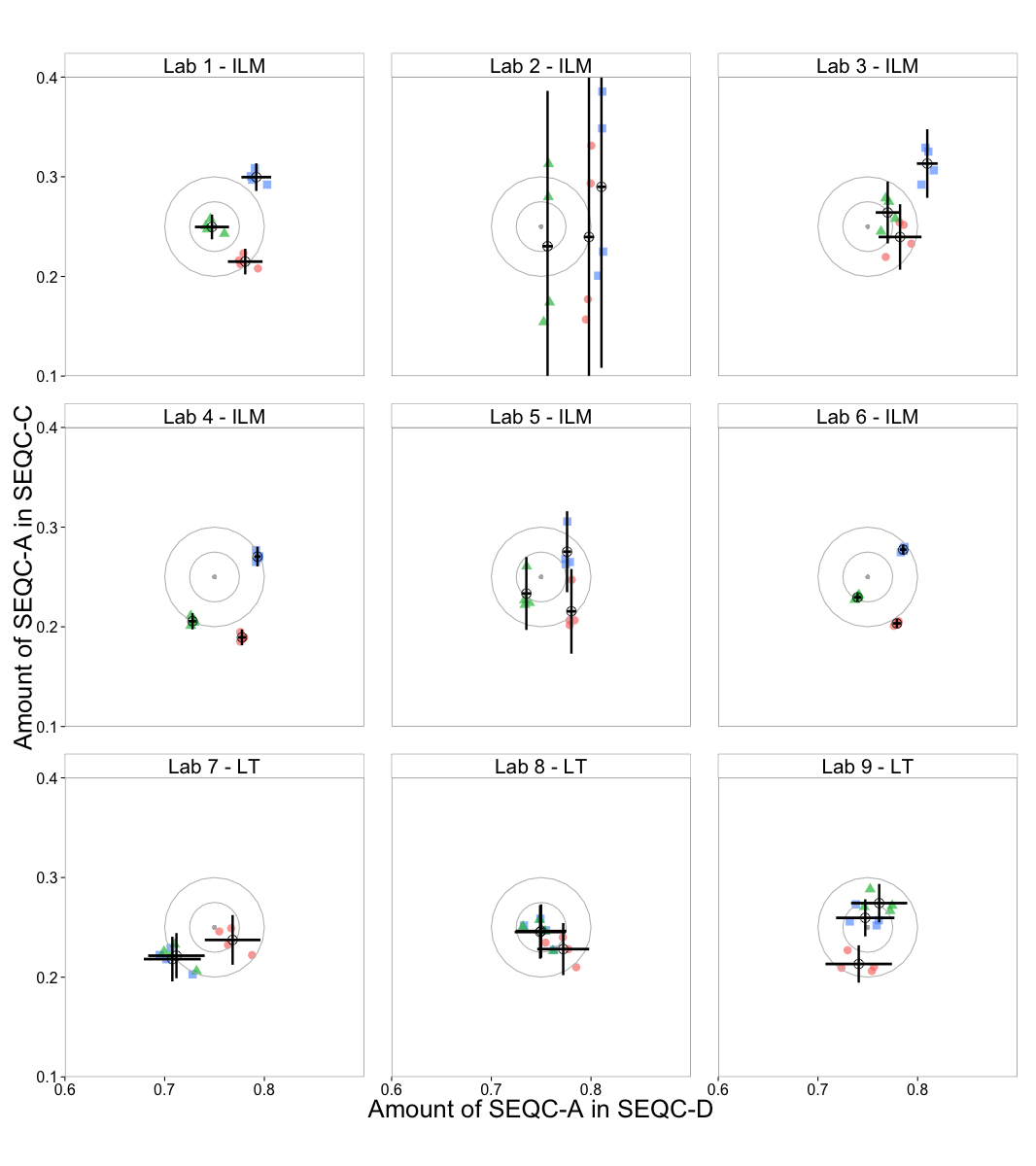

Supplement: Additional file 4: Table S1. — Enrichment fraction (ρ) calculations as a function of spike amount. Spike mass is accounted for in the enrichment calculation. The spike-ins varied by amount (“u” or “d” samples) and content (pools ‘a’ or ‘b’) in both tissue mixtures (1 and. 2). Calculated enrichment fractions vary by +/- 6 % across these 10 BLM mixtures, showing that the calculation is robust to spike-in mass and content. Enrichment calculations for the ERCC pools must account for the 3-plex nature of the mixes. The shown ratios are for the subset of spike-ins which are present at a 1:1 ratio in each sample. (PNG 119 kb) [file 12864_2015_1912_MOESM4_ESM.png]
