# Supplementary material for: Using mixtures of biological samples as process controls for RNA-sequencing experiments
Source: BMC Genomics. 2015 Sep 17;16(1):708. doi: 10.1186/s12864-015-1912-7 (PMC4574543; doi:10.1186/s12864-015-1912-7)
Supplement: Additional file 2: Figure S2. — The effect of using FPKM units. Estimates of enrichment fraction (light points are calculated using count values, dark points using FPKM values) result in a relatively poor solution to the mixture proportion. Both data types are taken from the same RSEM output. (DOC 29 kb) [file 12864_2015_1912_MOESM2_ESM.doc]

**Supplemental Table 1:** Enrichment fraction (*ρ*) calculations as a function of spike amount.   Spike mass is accounted for in the enrichment calculation. The spike-ins varied by amount (“u” or “d” samples) and content (pools ‘a’ or ‘b’) in both tissue mixtures (1 and. 2).  Calculated enrichment fractions vary by +/- 6% across these 10 BLM mixtures, showing that the calculation is robust to spike-in mass and content. Enrichment calculations for the ERCC pools must account for the 3-plex nature of the mixes. The shown ratios are for the subset of spike-ins which are present at a 1:1 ratio in each sample.

|  | BLM1-a | BLM1-ad | BLM1-au | BLM1-b | BLM1-bd | BLM1-bu | BLM2-a | BLM2-b | BLM2-bd | BLM2-bu |
| --- | --- | --- | --- | --- | --- | --- | --- | --- | --- | --- |
| Count Ratio | .0695 | .0095 | .6698 | .0719 | .0098 | .6342 | .0706 | .0737 | .0098 | .6649 |
| Spike Added | .08 | .01 | .64 | .08 | .01 | .64 | .08 | .08 | .01 | .64 |
| message fraction **ρ** | 1.152 | 1.058 | .955 | 1.112 | 1.017 | 1.009 | 1.132 | 1.085 | 1.025 | .962 |
