# Supplementary material for: Using mixtures of biological samples as process controls for RNA-sequencing experiments
Source: BMC Genomics. 2015 Sep 17;16(1):708. doi: 10.1186/s12864-015-1912-7 (PMC4574543; doi:10.1186/s12864-015-1912-7)
Supplement: Additional file 5: Note S1. — RNA-seq is capable of making transcript isoform-specific measurements. However, long reads of high depth are required to adequately differentiate between isoforms. Investigations of isoform-level measurements from the BLM dataset, which utilized 75 × 35bp paired-end reads on the 5500 and 100 × 100 bp paired-end reads on the HiSeq, showed that while the model is extensible towards such measurements, the reduced mean read counts make transcript isoform-level expression measurements less precise due to shorter read length and lower sequencing depth. 92 % of genes were modeled to within 1 log2 unit of the measured value, while only 85 % of transcripts were [38]. (DOC 28 kb) [file 12864_2015_1912_MOESM5_ESM.doc]

**Supplemental Note 1:**

RNA-seq is capable of making transcript isoform-specific measurements.  However, long reads of high depth are required to adequately differentiate between isoforms.  Investigations of isoform-level measurements from the BLM dataset, (Table 2) which utilized 75x35bp paired-end reads on the 5500 and 100x100bp paired-end reads on the HiSeq, showed that while the model is extensible towards such measurements, the reduced mean read counts make transcript isoform-level expression measurements less precise due to shorter read length and lower sequencing depth.  92 percent of genes were modeled to within 1 log2 unit of the measured value, while only 85 percent of transcripts were.

|  | Genes Measured | Genes Modeled (+/- 1 log2) | Percent | Transcripts Measured | Transcripts Modeled (+/- 1 log2) | Percent |
| --- | --- | --- | --- | --- | --- | --- |
| **BLM** | 19036 | 17641 | 92.6 | 23182 | 19772 | 85.3 |
| **SEQC** | 23947 | 22820 | 95.3 | 40333 | 38434 | 95.3 |

The substantially increased read depth in the SEQC experiment led to 95% of both isoforms and genes being consistently modeled.  In the SEQC dataset, 95% of detected isoforms could be consistently modeled to within a factor of 2, and the same percentage of genes could be reasonably predicted.  After applying a variance-stabilizing transformation using DEseq[38], every gene and transcript (100%) in the SEQC dataset were correctly modeled by these criteria.  The BLM dataset does not contain sufficient replication for variance-stabilizing analysis.
